# On the Non-Mercurial Treatment of Syphilis

**Published:** 1846-12

**Authors:** 


10. *On the Non-Mercurial Treatment of Syphilis.* By Dr. Scott.—Dr. Scott made some observations on the importance which attaches to the history of Syphilis. No subject could be more full of interest, or prove more clearly the necessity of strict investigation into what are considered the most established drnoctrines in medicine. Thirty years since there was no doctrine in the profession which was considered to be so well founded as the treatment of syphilis by mercury. In England none presume to differ from the opinion of John Hunter, that the disease was incurable without mercury, and not only that the medicine was required to remove the disease itself, but that to cure the disposition to it, and to secure the constitution from its ravages, an extended course of mercury was required. Sir Benjamin Brodie still retains this opinion; and Dr. S. observed that he would not have probably called the attention of the Society to this subject, had he not observed, in the lately published Essays by Sir Benjamin, some remarks, which, from so high an authority, appeared calculated to lead to what appeared to him an injurious line of practice. Every now and then a dissenting voice had been raised against the mercurial doctrine, but the profession in general adhered to the opinion of John Hunter.

Heberden considered it as one of the four specifics discovered in medicine. Allusion was made to the remarkable paper of Dr. Fergusson in the Med. Chir. Trans. of 1813, and the observations made by him on the disease, as it appeared in Portugal, and the opinion of the German physicians.

Sir Benjamin Brodie, in mentioning the work of Mr. Abernethy on Pseudo-Syphilis, considers that the illogical conclusions and extraordinary assumptions contained in it, have much diminished the value of this part of his writings. This work of Mr. Abernethy Dr. S. considered a most useful one, as having led the way to the investigation, from which such important results have been derived. Dr. S. then related his personal experience. In 1813 he was placed for a short time in Colombo, in charge of the venereal wards, in which the cases were all treated with mercury. Many of them he found were well in a few days, others in five or six, others in three weeks; periods too short to warrant the conclusion that they were venereal; they were therefore set down as cases of pseudo-syphilis. The number of these cases increased with the field of experience, and in a few years the use of mercury was gradually resigned in almost every case of local disease.

The secondary symptoms were few and slight, and never required an extended course of mercury. The same plan of treatment was also adopted with them, and in a few years Dr. Scott, then garrison surgeon at Point de Galle, entirely abandoned the use of mercury. The inference which he drew, however, was, not that the venereal disease was curable without mercury, but that the real disease did not exist in Ceylon. Dr. S. then described the miserable victims who were constantly found in military hospitals at that time, affected by extensive ulcerations, nodes, &c., who furnished a considerable number of the invalided, and many deaths. Since mercury was abandoned, such cases have disappeared from the hospitals. In 1818 and 1819, Dr. Scott became acquainted with the results of the investigation which had been carried on in England, and since that time had entirely abandoned the use of mercury as a specific. He had found many cases in which it was required as an alterative. After some remarks on laryngeal ulceration, diseases of the bones, &c., which are still met with in practice, Dr. S. stated that he considered every case of local disease curable without mercury, and that under such treatment the secondary symptoms, when they did occur, were slightly and easily managed. In fact, the disease ran a certain course, modified by peculiarities of constitution, and required only the treatment adapted to such modifications. Dr. S. drew a contrast between two cases of secondary symptoms which had been under his care at the same time, of young men of the same age, and of irritable and unhealthy constitutions. Both were severe cases, but in one the patient recovered in two months, while the other, after many narrow escapes, could only be pronounced cured after the lapse of a year from the first attack.

Dr. Maclagan expressed his satisfaction that Dr. Scott coincided in the views Dr. M. had long entertained on this subject. His confidence in mercury as a specific in syphilis had been first shaken when, after he was a graduate in medicine, he attended for some months the Lock Hospital in London, under Mr. John Pearson. There, every variety of form in the disease presented itself, but in very many cases seemed to be aggravated, rather than benefited, by the mercurial treatment; and though Mr. Pearson, in his lectures, and in his conversations with his more advanced pupils, still advocated the necessity for mercury in the cure of syphilis, he often expressed his doubts whether in many constitutions the use of mercury had not been more injurious than beneficial. While afterwards serving with the army in the Peninsula, and in charge of a Portuguese brigade, he had also been much struck with the apparent success which attended the treatment of the primary forms of the disease in the Portuguese soldier, by topical remedies alone, or merely with the additional use of Lisbon diet and drinks, and sometimes without either. He saw none of those cases of secondary symptoms in an aggravated form, to which his late lamented friend, Dr. William Fergusson, has alluded in his paper in the Transactions of the Medico-Chirurgical Society of London; but Dr. M. was then disposed to attribute the success of the non-mercurial treatment among the Portuguese to some peculiarity in the climate, and in the constitution and habits of the natives, which he afterwards had occasion to remark in a very different disease, Traumatic Tetanus, which, with few exceptions, assumed a less fatal form among the Portuguese wounded than among the British. On his return to Edinburgh, after the peace, Dr. M.'s attention had again been directed to the subject by the opinions long expressed by his early teacher Prof. Thomson, and by the opportunities of seeing the practice in the Depôt Hospital in Edinburgh Castle, under Dr. Thomson's charge, as well as in that, and in Regiment Hospitals, under Dr. Hennen, Mr. Johnston, and Dr. Bartlett of the 88th regiment, the latter of whom published an excellent Thesis, at his graduation, on the non-mercurial treatment. This treatment had also been adopted in the practice of Staff-Surgeon Guthrie, and in that of Mr. Rose of the Coldstream Guards, and since very generally and successfully throughout the army. Since 1818 Dr. M., with a few exceptions where the patients' scruples afford full explanation, demanding its modified use, has adhered to the non-mercurial plan of treatment both in dispensary and in private treatment, and in no one instance has had reason to regret it. Many who were then so treated are his patients still, fathers of families enjoying, as well as their offspring, excellent health, and without the occurrence in the period that has elapsed of any secondary symptoms of an aggravated form. On the other hand, he has seen too many cases where the use of mercury to its full extent has been productive of constitutional injury of the most serious character.

Dr. D. Maclagan alluded to the success which attended the practice of Dr. Fricke in Hamburg, and Professor Krukenberg in Halle, in corroboration of the benefits of a non-mercurial system of treatment.

Dr. Bennett stated, that the last account of Dr. Fricke's practice, with which he was acquainted, is to be found in Sir Alexander Crichton's Commentaries on Medicine. This treatment had been tried on a large scale in the various garrisons in France, Germany and Sweden, and reports had been given to the various governments, amounting altogether to upwards of 80,000 cases, the general results of which were quite in accordance with the experience of Dr. Scott. He thought that one of the best evidences of the non-mercurial treatment existed in the fact, that those dreadful secondary and tertiary cases which were formerly so common, are now seldom met with, and that pathological specimens of syphilitic bones, although common in museums, are at present scarcely to be obtained.

Dr. R. Mackenzie was of opinion that the observations which had been made were directed rather against the abuse than the use of mercury. As surgeon to the Lock Hospital of Edinburgh, he had seen many cases where the sores, however obstinate, had at once improved in character as soon as the constitution was affected with the drug. He alluded to two cases especially, in which this was observed, where mercury was given for iritis, but in which obstinate chancres on the genitals also began to heal as soon as the medicine produced its physiological effects.

Dr. A. D. Campbell stated that mercury was also necessary in the syphilitic eruptions of children.—*N. Y. Journal of Med. and Collateral Sciences.*

